# Participant Experiences of Therapeutic Touch in Psilocybin‐Assisted Therapy

**DOI:** 10.1002/brb3.71262

**Published:** 2026-02-16

**Authors:** Rachel Ham, John Gardner, Adrian Carter, Paul Liknaitzky

**Affiliations:** ^1^ Department of Psychiatry, School of Clinical Sciences Monash University Melbourne Australia; ^2^ School of Psychological Sciences Monash University Melbourne Australia; ^3^ School of Social Sciences Monash University Melbourne Australia; ^4^ Monash Bioethics Centre, School of Philosophical, Historical and Indigenous Studies Monash University Melbourne Australia

## Abstract

**Introduction:**

Although commonly used in psychedelic‐assisted therapy, the role of therapeutic touch remains loosely defined and ethically sensitive. Gaining insight into how participants experience and interpret touch during psychedelic sessions is essential for informing safe and effective clinical practice.

**Methods:**

Participants were sampled from a large randomized clinical trial of psilocybin‐assisted therapy that permitted protocol‐defined supportive touch. Longitudinal qualitative data (39 semi‐structured interviews) were analyzed from n = 18 participants. Interviews covered expectations, experiences, and reflections on the use of touch during acute psychedelic states, before and after dosing. Thematic analysis was used to identify major themes.

**Results:**

Participants expressed varied preferences and responses to therapeutic touch. Most valued its availability, particularly after firsthand experience, describing its capacity to foster emotional connection, provide grounding during intense affective states, and modulate the depth of psychedelic experience. Several reported perceiving therapeutic benefit directly attributable to touch. Acceptability was consistently linked to the quality of the therapeutic relationship and robust consent processes. Some participants also identified potential for discomfort or distraction, underscoring the need for sensitivity to individual history and context.

**Conclusions:**

Therapeutic touch may support emotional safety and affect regulation during acute psychedelic states. Findings highlight the importance of explicit preparation, consent, and attunement when incorporating touch into psychedelic therapy. Further research should inform therapist training, individualized consent frameworks, and safety protocols to guide ethical and effective use in clinical practice.

## Graphical abstract

Touch can enhance subjective safety and affect regulation in acute psychedelic states when utilised based on individual fit, with consideration of its. potential and risks, and within formal consent processes.

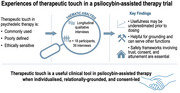

## 1 Introduction

Psychedelic‐assisted therapy (PAT) is emerging as a promising mental health intervention (Bahji et al. 2020; Carhart‐Harris et al. 2021; Garcia‐Romeu et al. 2014; Goldberg et al. 2020; Griffiths 2016; Moreno et al. 2006). In 2023, the Australian Therapeutic Goods Administration down‐scheduled psilocybin and MDMA, allowing use under strict conditions by authorized psychiatrists and their teams (Therapeutic Goods Administration 2023). As clinical implementation of PAT expands, there is a pressing need for evidence‐based guidelines around PAT therapeutic techniques. This paper examines one key aspect of current PAT practice: therapeutic touch.

### 1.1 History of Touch in Psychotherapy

Touch in psychotherapy has a complex history, originating with Freud who initially used touch but later discouraged it, emphasizing analytic neutrality in the “talking cure” of psychoanalysis (Ben‐Shahar 2018; Phelan 2009). Modern psychotherapy largely defines itself as a “non‐touching profession” (C. Harrison et al. 2012). While sexual contact is prohibited (e.g. Australian Psychological Society [APS] 2016a), guidelines on other forms of touch (e.g. task‐oriented, attentional, celebratory; see Hunter and Struve 1998 for a taxonomy) are sparse beyond limited recognition of the value of reassuring gestures like handshakes or arm touches (APS 2016b, 2016c). Advocates of physical touch in psychotherapy stress its importance in human development and attachment, healing, and strengthening therapeutic relationships (Durana 1998). Modalities such as somatic therapy, Gestalt therapy, and eye movement desensitization and reprocessing therapy employ touch (Hase 2021; Hunter and Struve 1998; Kepner 2001; Kuhfuß et al. 2021), but robust empirical research is lacking. Contemporary PAT trials share features with standard psychotherapies—therapeutic alliance, supportive environments, and client engagement (Gründer et al. 2023; Gukasyan and Nayak 2022; Wolff et al. 2024). However, PAT protocols also introduce unique considerations tied to psychedelic dosing sessions.

### 1.2 Supporting People During Psychedelic Experiences

Therapeutic touch in PAT typically proposes to enable therapeutic support where verbal communication is either not possible or risks undermining inner‐focus during the psychedelic experience. Psychedelics can profoundly alter awareness (Hirschfeld and Schmidt 2020) that can be psychologically challenging (Barrett et al. 2016), making psychotherapeutic support essential during dosing sessions (Johnson et al. 2008). Yet psychedelics transiently impair linguistic capacity (Wießner et al. 2023) and induce “indescribable” experiences (Griffiths et al. 2016) that risk being disrupted by communication attempts (Sanz et al. 2021; Wießner et al. 2023). Accordingly, non‐conversational therapeutic strategies may be needed during acute psychedelic effects. Some clinicians consider touch a useful non‐verbal tool, but empirical evidence and research into participants’ attitudes are scarce. This study addresses that gap.

Touch was used since the first wave of PAT (1950s–1970s). While some clinicians advocated maternal‐style physical holding (Martin 1957), others cautioned against non‐essential contact during altered states (Buckman 1967). Current PAT manuals permit physical touch (Guss et al. 2020; Haden 2019; Mithoefer 2017), yet definitions range from minimal distress support (e.g., handholding) to somatic‐style interventions (Kuhfuß et al. 2021; Rosendahl et al. 2021). Despite increasing clinical and public interest in PAT (Luoma et al. 2023; McLane et al. 2021), and a growing literature on the ethical delivery of therapeutic touch (Neitzke‐Spruill et al. 2025), the safety, utility, or acceptability of touch in PAT to participants has not been directly investigated.

### 1.3 Ethics of Touch in PAT

The use of touch in PAT raises significant ethical considerations. These include consent‐related power imbalances; the risk that touch is experienced as sexual or boundary‐violating; professional boundary consequences; and cultural or personal differences (T. R. Harrison 2023; Simon 1999; Smith et al. 1998; Zur 2007, 2). High‐profile misconduct cases in PAT amplify these concerns (C. Harrison et al. 2012; Buisson, in Kay Ross and Nickles 2023), as do reports of increased dependency on therapists (Kay Ross and Nickles 2023). Another ethical concern is whether patients can meaningfully consent to touch while under the influence of consciousness‐altering drugs. Acute psychedelics effects include increased confusion, transient cognitive‐perceptual distortion, increased suggestibility and increased meaning‐making (Carhart‐Harris et al. 2015; Grahl Johnstad 2021; Hartogsohn 2018; Kaelen et al. 2015; Preller et al. 2017). These characteristics may increase the risks that touch causes discomfort or is misinterpreted. Attempts to address these risks include the use of therapist dyads and formal consent protocols (McLane et al. 2021; Harlow, 2013, as cited in Passie 2018).

Given its prevalence, vague definition, and ethical complexity, understanding how participants experience therapeutic touch is critical. This study explores these perspectives qualitatively in a large psilocybin‐assisted therapy trial for Generalized Anxiety Disorder (GAD) by asking:

1. How do participants view therapeutic touch in PAT?
2. Do these views change over the course of treatment?
3. What is the perceived utility of therapeutic touch in PAT?

## 2 Methods

### 2.1 Clinical Trial

This research was approved by the Monash University Human Research Ethics Committee. Data were collected from a randomized clinical trial (Psi‐GAD‐1) of PAT for severe GAD (for study details see ACTRN12621001358831). Treatment included two dosing sessions (psilocybin 25–30 mg or diphenhydramine 75–100 mg) and nine psychotherapy sessions with a mixed‐gender co‐therapy team. Drug administration sessions followed standard clinical trial procedures, including individual dosing in a comfortable university laboratory setting, under the supervision of a co‐therapy team.

The trial permitted minimal, supportive touch (e.g., handholding) during dosing sessions, mainly for managing distress. Non‐contact supports included weighted blankets and hot water bottles. Somatic psychotherapy was not permitted; sexual or sensual touch was prohibited. Consent was established and practiced before dosing, and participants could adjust their preferences between sessions. This protocol aimed to uphold boundaries and promote a safe, respectful therapeutic environment. All trial participants consented to therapeutic touch with personalized specifications, and all but one participant analyzed qualitatively received therapeutic touch.

### 2.2 Data Collection and Analysis

**FIGURE 1:**
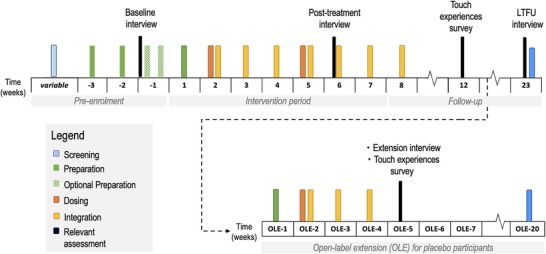
Treatment and relevant assessment timeline for participants in the PsiGAD‐1 Clinical trial. *Note*: LTFU = long term follow‐up; OLE = open label extension.

This study used qualitative and descriptive data collected via survey and interview (Figure 1).

#### 2.2.1 Descriptive and Survey Data

Demographics were collected at screening via REDCap (Harris et al. 2019) for all Psi‐GAD‐1 participants. Six weeks after the second dosing, participants rated: *In your view, how important is the role of therapeutic touch in psychedelic‐assisted psychotherapy?* (Visual Analogue Scale; 0 = *Not at all important* to 100 = *Essential*). Placebo‐group ratings were collected at three weeks after their extension‐arm dose. Responses were summarized descriptively and visualized to show diversity.

#### 2.2.2 Qualitative Data

Semi‐structured interviews (∼60 min) were conducted via Zoom at three time‐points by the first author and another trained qualitative researcher. Participants were informed that the interviews aimed to understand their personal experiences of the treatment. Interviews explored treatment experiences, including expectations and experiences of therapeutic touch. Interviewers acknowledged their interest in the topic and mitigated bias through reflexive practice. Recordings were transcribed and coded in NVivo.

Qualitative data came from 39 interviews across 18 clinical trial participants (13 active; 5 placebo) from 62 eligible trial participants (94.5% consented; 84.93% completed). Data were purposively selected based on two criteria: (1) depth of description, including interviews that provided detailed, reflective accounts suitable for thematic analysis, and (2) clear relevance to the research question, ensuring included material directly addressed participants’ experiences of therapeutic touch in psychedelic‐assisted therapy. Ratings on the importance of the touch scale were also considered to capture a variety of perspectives. Sampling continued until thematic saturation was reached (Guest et al. 2006). Demographic information for the full and qualitative samples appears in Table 1.

**TABLE 1:** Demographic information for the whole sample and qualitative subsample.

Variable	Whole sample (*n* = 73)	Qualitative subsample (*n* = 18)
**Age (m, SD)**	39.73 (10.54)	38.12 (11.09)
**Gender**		
Man	28 (38.4%)	6 (33.33%)
Woman	42 (57.5%)	11 (61.11%)
Non‐binary	2 (2.7%)	1 (5.56%)
Self‐described	1 (1.4%)	1 (5.56%)
**Education**		
Not completed high school	1 (1.4%)	1 (5.56%)
High school	11 (15.1%)	3 (16.67%)
Undergraduate degree	29 (39.7%)	6 (33.34%)
Postgraduate degree	32 (43.8%)	8 (44.44%)
**Place of birth**		
Oceania	59 (80.82%)	16 (88.89%)
Europe	5 (6.85%)	1 (5.56%)
North America	4 (5.48%)	0
Asia	3 (4.11%)	1 (5.56%)
Africa	2 (2.74%)	0
**Ethnicity**		
Aboriginal Australian	1 (1.37%)	1 (5.55%)
Non‐indigenous Australian	55 (75.34%)	14 (77.78%)
Non‐Australian	17 (23.29%)	3 (16.67%)
**Importance** **of** **touch** **ratings** **(** ***m***, **SD** **)**	75.38 (21.12)*	78.94 (15.12)
*Note: response from n = 72 participants, with 1 withdrawal prior to datapoint		

Thematic analysis (Braun and Clarke 2006) was inductive and iterative. A subset (*n* = 3) of interviews was triple coded by authors RH, PL, and AC. After reaching consensus on themes, the first author, who has a clinical psychology background and approaches this work with an awareness of ethical concerns surrounding boundary violations in PAT, completed coding. Codes were reviewed for internal coherence and validated with the team. To capture diverse experiences, interviews with atypical, contradictory, or negative accounts of touch were prioritized. Three primary themes emerged (Table 2).

**TABLE 2:** Qualitative analysis: themes and subthemes.

Theme	Sub themes
**Expectations and attitudes towards therapeutic touch**	Initial expectations of touch
	Shifting attitudes towards touch following the psychedelic experience
**Varied experience and interpretation of touch**	The role of touch‐facilitated connectedness for intense emotional experiences
	Bridging worlds: touch can facilitate or hinder the ‘depth’ of psychedelic experience
	Touch considered therapeutic, independently of its support of psychedelic experience
**Relational dynamics in delivery: trust and transparency**	The process of consent
	The role of trust and attunement in the experience of touch

Thematic analysis (Braun and Clarke 2006) followed an inductive and iterative coding process. A subset (*n* = 3) of interviews was triple coded (by authors RH, PL, and AC). Once the coding team reached consensus on themes, the remaining analysis was completed by the first author. To avoid premature saturation, interviews discussing atypical, contradictory, or negative experiences with touch were prioritized for analysis. Codes were regularly examined for internal coherence and validated with the team. Three related but distinct primary themes were developed.

## 3 Results

### 3.1 Survey Results

**FIGURE 2:**
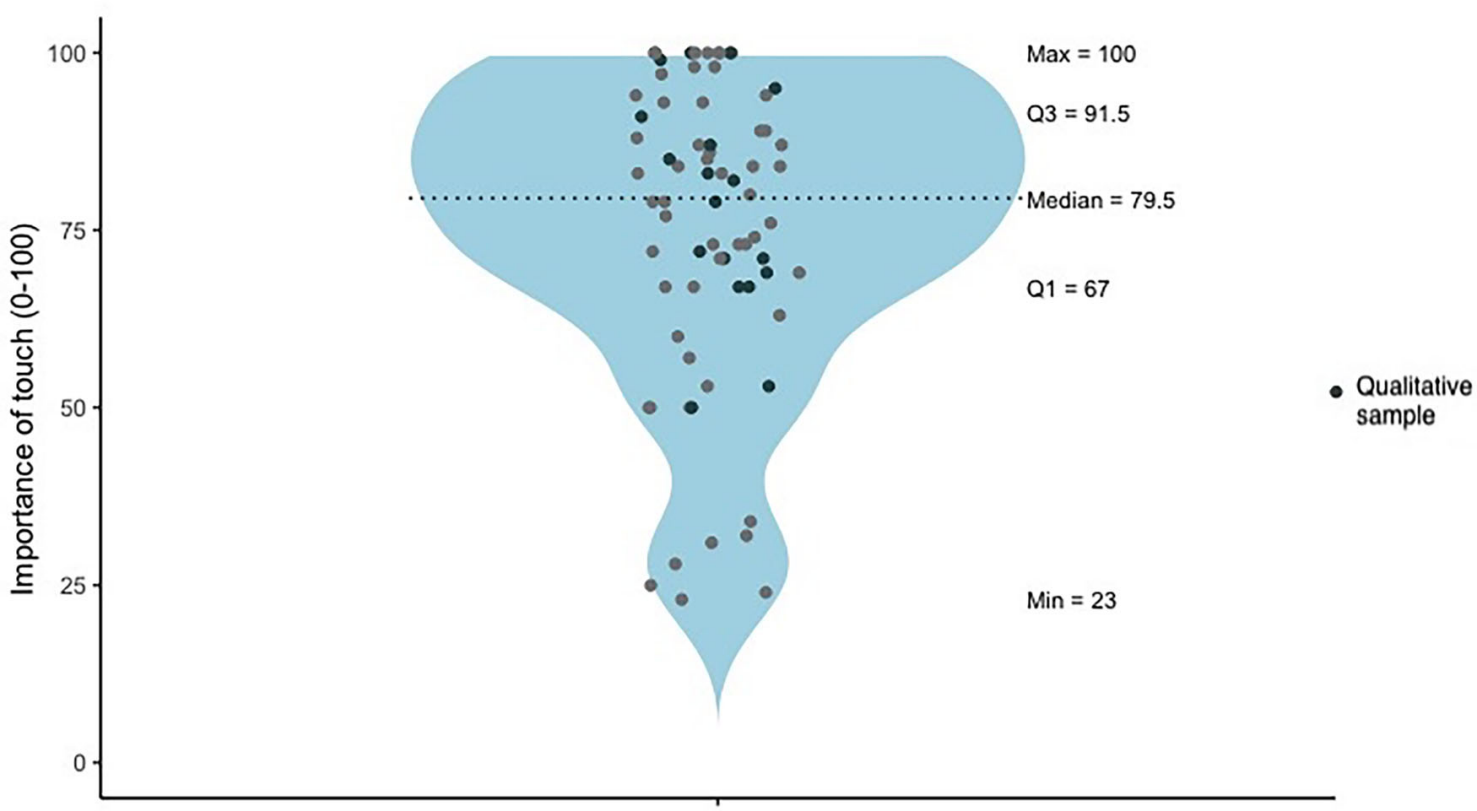
Participant ratings of the importance of touch in psychedelic‐assisted therapy.

Participant ratings of the importance of therapeutic touch for *n =* 72 participants (following psilocybin administration during the control phase or open label extension) are presented in Figure 2.

### 3.2 Qualitative Results

#### 3.2.1 Theme 1: Expectations and Attitudes towards Therapeutic Touch

##### 3.2.1.1 Initial Expectations of Touch

Prior to dosing, participants’ attitudes towards the use of touch in PAT were generally positive or neutral. Participants commonly identified distress management as a potential application of therapeutic touch and thought that touch would be “grounding” or “calming” in moments of distress.

*“For me, [touch] is a way of feeling safe and feeling connected … I think it's a very human thing. It would make sense to me that if I'm in that position where I'm feeling quite vulnerable … quite out of myself, that [touch] is there to potentially help ground me and help me feel safe.”*

Participant 1, Woman, Baseline

Many participants linked their expectations about touch in PAT to how they generally felt about physical contact in other social contexts. For example, those identifying themselves as “huggers” expressed more positive expectations. Those who described themselves as being generally less comfortable with touch occasionally cited difficult or confusing touch experiences during childhood, including strained relationships with parents or other adults.

*“…you would typically have like therapeutic touch … when you were a child, like, your mother kind of comforting you, patting you on the back kind of thing. And my childhood wasn't like that.”*

Participant 2, Woman, Baseline

Reasons given to explain participants’ hesitancy to incorporate touch into their therapy session included concerns about touch being a disconcerting sensory experience if unexpected or anticipating that touch would make them feel self‐conscious (e.g. about being sweaty).

##### 3.2.1.2 Shifting Attitudes Toward Touch Following the Psychedelic Experience

After psilocybin dosing, many participants described therapeutic touch as more important than expected, often with surprise. Several participants stated that they wouldn't want to go through a psychedelic experience without having touch available to them.

*“Oh, man, the touch was such a major part of this. Like, more than I ever thought it would be.”*

Participant 1, Woman, Post‐treatment

Of those participants who raised initial concerns or described themselves as less comfortable with touch generally, some went on to find touch useful.

*“They would like, put a hand on my shoulder when I needed it ‐ and I was so afraid of being touched ‐ and then it was fine … and it was so fine that like, when they went away, I could be like “no, no, no, come back” ((laughs)) … That's new.”*

Participant 4, Non‐binary, Post‐treatment

An extension arm participant contrasted the experience of touch with and without psilocybin:

*“[Touch] probably felt a bit uncomfortable during the [placebo] dosing sessions … just because it's not something you normally do with your therapist, especially. But that [discomfort] was probably not there with the psilocybin because I think it's a really strong medicine and you just have this like great empathy and connection with everything”*

Participant 3, Man, Post‐Extension

Not all participants experienced this shift in attitudes. Several participants expressed ambivalence about the use of touch, and not all participants utilized touch during their treatment. Some expressed a lack of reliance on touch but noted that it was “good to have the option” (Participant 4).

*“they [used touch] a little bit at the end … it wasn't, like, helpful or unhelpful … I didn't benefit from it too much. But it didn't feel uncomfortable … I think at that stage, [the effects of the psilocybin] had probably worn off a little bit.”*

Participant 5, Woman, Post‐Extension

#### 3.2.2 Theme 2: Varied Experience and Interpretation of Touch

##### 3.2.2.1 The Role of Touch‐Facilitated Connectedness for Intense Emotional Experiences

Participants utilized therapeutic touch for diverse emotional experiences. This predominantly included distress management (e.g., during experiences of fear, loneliness, emotional overwhelm, and paranoia in dosing sessions). One participant described a transient drug‐induced hallucination in which he experienced his therapists calling him a “monster”, but described the usefulness of physical presence and touch when verbal support was compromised:

“*We were sitting on the floor in a circle and like, we had [moments] where we were hugging … It was like they were on the same journey with me. So yeah, they were super, super helpful … it was almost irrelevant, the talking part, but them being there was obviously good … even though [during a transient drug‐induced hallucination] they're telling me, ‘yeah, you're a monster’, I was still hugging ‘em and telling’ em I love em and stuff*.”

Participant 6, Man, Post‐Extension

Touch was also sometimes employed by participants to amplify positive emotions of joy or euphoria:

“*I was just looking at those clouds and of course they were making all these different shapes and the colours were changing … I actually reached out for [Therapist One] and [Therapist Two] because I really wanted them to be part of it. So I took their hands and, and I had this feeling of, I suppose, euphoria. Like, a childlike, kind of, giddiness*.”

Participant 1, Woman, Post‐treatment

Many participants said touch fostered connection, making difficult emotions more manageable and positive ones more enriching. For some, this connection was felt towards their therapists specifically, while others described a more diffuse or general sense of connection (e.g., not being alone in their minds, a sense of not being alone in the world generally, or a sense of shared humanity). However, one participant reported that touch amplified their difficult emotional experience:

*I was really quite emotional. And when one of the two grabbed my upper arm and held it, that emotion got a lot stronger straightaway … Later [when touch was offered again] I paused and then declined because I couldn't deal with that level of emotion at the time*.

Participant 7, Man, Post‐treatment

##### 3.2.2.2 Bridging Worlds: Touch Can Facilitate or Hinder the “Depth” of Psychedelic Experience

Participants differentiated an immersive inner world when using the eye mask and headphones (“going in”) compared to removing these sensory tools (“coming out”). Participants described strategically using touch to manage the distress or overwhelm experienced within the inner world without having to disengage, in some cases allowing people to ‘go deeper’ or ‘stay longer’:

*“It was kind of like two different places, two different existences, really. So having that therapeutic touch meant I could stay in… but know that like, the other place existed ((laughs)) if you will. So it just meant that I could still continue doing the work of the session … and feel … that reassurance. So it didn't have to be one or the other. It could kind of bridge that a little bit”*

Participant 2, Woman, Post‐treatment

Some participants expressed concern that touch could also be distracting. Although many evaluated touch as less distracting than verbal support, some acknowledged that touch had the potential to disrupt meaningful therapeutic work by drawing someone “out” of their internal world, or away from necessary “surrender”:

*“Touch kind of like, brings you out … I was probably a little bit upset, but I said, ‘thanks for leaving me in there’ … It sort of changes everything, the touch. So yeah, just having that knowledge, you know, if someone's having like an experience where they really need to be in … I guess, and that the touch can bring you out of the experience and that can be a negative thing. Could be a negative thing … or a positive thing.”*

Participant 3, Man, Post‐Extension

##### 3.2.2.3 Touch as an Agent of Therapeutic Insight

Participants constructed the meaning and purpose of touch in nuanced ways shaped by their self‐views, histories, and treatment goals. Many reported that touch facilitated progress towards broader therapeutic offering chances to practice challenging interpersonal processes such as asking for help, releasing dysfunctional independence, and embracing connection. In these ways, touch was viewed as a meaningful treatment element beyond supporting the psychedelic experience. For some, it was one of several “acts of service” provided by therapists during dosing:

*“I've always been kind of hyper independent and I've started asking for stuff more. Asking for help more … I think that's all come from [Therapist One] and [Therapist Two] creating an environment where I'm able to reach out and take their hand. Or I'm able to say, ‘can you pass me the water? Or can I have a snack? Or can you get me the blanket?’”*

Participant 1, Woman, Post‐treatment

#### 3.2.3 Theme 3: Relational Dynamics in Delivery: Trust and Transparency

##### 3.2.3.1 The Process of Consent

The therapeutic touch protocols used in this trial were generally evaluated positively by participants. All participants described the therapeutic touch processes as clear and professional, although they suggested extending consent to non‐therapeutic touch (e.g. touch to the shoulder to get their attention while wearing headphones). Some participants highlighted that the explicit consent process had provided feelings of safety around the use of touch.

*“It's been really nice … the way that they spoke about [touch] really transparently today, and like, consent and stuff, it's made me feel really safe about it. And very clear kind of guidelines about like they won't touch between the neck and the knees, unless it's on the arms and that sort of thing. It's been really helpful.”*

Participant 8, Woman, Baseline

One participant described the trial's touch protocols as showing “excruciating carefulness.” Some felt the protocols were excessive, either in how often touch was discussed or in the level of detail (e.g., specificity around whether a handhold or a rub on the shoulder felt more appropriate to participants). Most qualified these statements by acknowledging the function of these protocols in protecting the safety of participants, or the reputation of therapists or psychedelic research.

##### 3.2.3.2 The Role of Trust and Attunement in the Experience of Touch

Many participants emphasized trust in the therapist‐client relationship as key to therapeutic touch. Relevant factors included the co‐therapy dyad and therapist gender. One participant felt two therapists enhanced safety but suggested this might be unnecessary with a trusted long‐term therapist. Two female participants initially requested touch only from the female therapist but changed their preferences to allow both therapists to provide touch in the following dosing session as trust developed. Notably, several participants who were initially hesitant described their therapists as “strangers” during baseline interviews:

*“It felt like [therapeutic touch] was probably more helpful than I would have imagined. At first, I was struggling to understand that I could even really feel safe with these kind of strangers in the room, but it was like … over time developing that kind of relationship with them, it did feel helpful.”*

Participant 8, Woman, Post‐treatment

Participants also highlighted the need for therapeutic attunement for touch to be delivered safely. They expected therapists to “read the room” (Participant 6) and respond to body language cues about touch preferences and appropriateness. Describing a moment of gratitude for his therapists’ management of touch, one participant explained how reflection during therapy sessions was utilized to optimize its appropriate use:

*“They didn't know [exactly when touch was needed]. In one of the integration sessions, they asked about it. And I said, ‘no, what you did was perfect’. They were like, ‘we don't want to leave you in there if you're really upset. But then we don't want to bring you out’. So it's like an impossible balance. And you'll probably never like, get it a hundred percent right. But um, maybe just with experience and stuff it'll come.”*

Participant 3, Man, Post‐Extension

Additional illustrative quotes and counterexamples for all qualitative themes are provided in Supplementary Table 1.

## 4 Discussion

This study is the first to evaluate the use of therapeutic touch in PAT using data directly from clinical trial participants. Findings highlight its potential value as a supportive tool when embedded within a comprehensive and robust consent process. Variation in responses underscores the importance of adopting an individualized approach. These results can inform guideline development, therapist training, and clinical integration of touch in PAT.

### 4.1 Participant Evaluations and Motivations

Participants’ engagement with touch was highly idiosyncratic, reflected in both qualitative and descriptive data. The high mean rating of touch importance (m = 75.38), and negatively skewed distribution (nine participants gave a maximum rating) suggest that most valued touch. Yet the wide range (min = 23; max = 100; see Figure 2) indicates that not all participants found touch important, reinforcing the need for tailored rather than universal protocols. Qualitative data similarly reflected generally positive attitudes alongside variability in expectations, utilization during dosing sessions, and perceived helpfulness.

Participants used touch to manage emotions, navigate psychedelic states, and pursue broader therapeutic goals. Although consistent with positive attitudes to touch in pure psychotherapy contexts (Horton et al. 1995), our findings suggest that touch may synergize with key aspects of the acute psychedelic experience. Participants described the usefulness of touch in managing heightened emotional intensity and a foreign state of consciousness, both key aspects of the acute psychedelic experience (Barrett et al. 2016; Hirschfeld and Schmidt 2020; Roseman et al. 2019). Findings also highlight challenges for the use of touch in PAT: while no participants reported significantly adverse effects, touch may distract from meaningful experiences, vary in appropriateness across the acute drug effect stage, or paradoxically intensify, rather than grounding, emotions. While PAT manuals regularly incorporate therapeutic touch (Guss et al. 2020; Haden 2019; Mithoefer 2017), few have characterized these benefits and challenges, which are critical for informing strategic clinical use.

### 4.2 Enhancing Consent and Trust

While some literature suggests that touch may strengthen the therapeutic alliance (Berendsen 2018; Horton et al. 1995), participants here emphasized the reverse—that a strong alliance was a prerequisite for touch. Establishing a safe, predictable setting through robust consent processes seemed to enhance confidence and trust, supporting both the use of touch and the broader therapeutic relationship, which in turn may enhance therapeutic outcomes (Grof, 1980; Phelps, 2017).

The positive shifts in some participants’ views of touch after psilocybin suggest they may initially underestimate its value in PAT. While ethical concerns have been raised about withholding touch during psychedelic states (Haden 2019; Mithoefer 2017), this did not arise in our sample, likely due to high consent rates supported by flexible consent procedures. High consent and positive attitudes are not surprising given favorable attitudes toward touch in psychotherapy (Tanzer et al. 2022), but may reflect sample‐specific traits (e.g., high trait‐agreeableness or complex attachment histories; Cervera‐Solís et al. 2022). Given the difficulty of predicting its value, inviting participants to predefine “emergency‐only” touch could ensure supportive options are available if needed (McLane et al. 2021). For those declining touch, non‐contact supports (e.g., weighted blankets, synchronized breathing, physical proximity) may offer similar benefits. Future work could examine views of those who do not receive touch, examine the individualized “journey” of touch experiences across dosing sessions, and investigate systems for informed consent and strategic use of touch.

Post‐dosing discussions about therapeutic touch, regardless of whether it was used, proved valuable. Touch constitutes a boundary crossing, as it departs from standard psychological practice (Aravind et al. 2012). One participant described a group‐hug during a difficult dosing experience, which, while positive for them, is potentially a higher‐risk application of touch. While such moments require clinical judgment, intuition alone cannot reliably ensure safety and should be seen as a last resort. Structured debriefing offers a harm reduction measure, helping to prevent boundary crossings from becoming boundary violations by clarifying consent, reinforcing autonomy, and prompting therapist reflection (Gutheil 2008). Similarly, informed consent should also address the potential for varied interpretations of touch under psychedelics.

### 4.3 Considerations and Future Directions

The benefits and challenges of therapeutic touch in PAT will likely vary by clinical indication, psychedelic compound, and therapeutic context. Participant perspectives on touch varied within and between participants, and similar variability is expected across other populations (e.g., those with trauma histories may have distinct needs; Strauss et al. 2019). Our study is limited by the same sampling homogeneity observed across PAT research (Haft et al. 2025); investigating diverse cultural frameworks surrounding touch is essential. Further research should also explore how touch interacts with faster‐acting (Strassman and Qualls 1994) or empathogenic compounds (De Wit and Bershad 2020); these results are specific to psilocybin.

Our study involved a minimalistic, client‐directed touch protocol with detailed consent, contrasting with approaches involving extensive bodywork or somatic methods (Haden 2019; Mithoefer 2017). While our findings justify therapeutic touch as a tool to foster support, connection, and grounding, we caution against treating it as an active intervention during acute psychedelic states until further evidence and ethical guidance are established. Protocols involving more intensive physical contact likely carry increased risks and should be implemented only with clear transparency, adequate training, and rigorous monitoring.

Future research should examine PAT therapists’ needs and challenges regarding touch. These insights could inform training programs for emerging practitioners and guide the safe, effective use of therapeutic touch, while addressing the ethical balance between therapist and patient autonomy.

## 5 Conclusion

In summary, touch in PAT appears to be one way to support psychological and emotional needs during acute psychedelic states, with acceptability linked to a strong therapeutic alliance. When paired with thorough preparation, integration, and a minimalistic, consent‐driven protocol, touch was viewed positively by many participants. Continued research into the nuanced role of therapeutic touch, accounting for individual differences and therapeutic relationship dynamics, is essential for optimizing PAT outcomes.

## Author Contributions

**Rachel Ham**: conceptualisation, methodology, validation, formal analysis, investigation, data curation, writing – original draft, writing – review – editing, visualization, project administration **John Gardner**: methodology, validation, writing – review and editing, supervision. **Adrian Carter**: conceptualisation, methodology, validation, writing – review and editing, supervision **Paul Liknaitzky**: conceptualisation, methodology, validation, resources, writing – review and editing, supervision.

## Funding

This research was funded by Incannex Healthcare Limited and Monash University, with Psilocybin provided by Usona Institute. The funder had no role in the design, data collection, data analysis, and reporting of this study.

## Conflicts of Interest

Dr Liknaitzky has received research funding from Incannex Healthcare Ltd, the Multidisciplinary Association for Psychedelic Studies, and Beckley Psytech, and is on the Scientific Advisory Board of the MIND Foundation, Germany. Incannex Healthcare Ltd contributed partial funding for this study. These organisations were not involved in any aspect of this paper, including the study design and conduct, the decision to write the paper, drafting the paper, or its publication. The remaining authors declared no potential conflicts of interest with respect to the research, authorship, and/or publication of this article.

## Ethics Statement

This study was conducted as part of the Psi‐GAD‐1 Clinical trial and was approved by the Monash University Human Research Ethics Committee (Project ID: 29947) on October 26, 2021. All participants provided written informed consent before participating, including for the publication of deidentified quotes from their interview transcripts.

## Supporting information




**Supplementary Table**: brb371262‐sup‐0001‐TableS1.pdf

## Data Availability

Due to the identifiable nature of the qualitative data, it has not been made publicly available. However, efforts have been made to ensure transparency by providing extensive illustrative quotes (including counterexamples) within the text and supplementary materials.
