# Supplementary material for: Participant Experiences of Therapeutic Touch in Psilocybin‐Assisted Therapy
Source: Brain Behav. 2026 Feb 16;16(2):e71262. doi: 10.1002/brb3.71262 (PMC12910122; doi:10.1002/brb3.71262)
Supplement: Supplementary file 1 — Supplementary Table: brb371262‐sup‐0001‐TableS1.pdf [file BRB3-16-e71262-s001.pdf]

**Supplementary Table 1**

*Summary of themes and relevant quotes*

| Theme and Subtheme | Relevant quotes |
|---|---|
| **<u>Expectations and evaluations of therapeutic touch</u>** | |
| 1.1. Expectations of touch | *"I can imagine if I was feeling distress, just to have that kind of grounding touch from someone else could be really helpful in the moment just to help calm me down."* |
| | *"I was molested, right. [If] I was like, taking drugs, maybe [touch] would trigger me in some way. But I don't imagine it would. I think [verbal support] would be more beneficial, just guessing … I'm not probably, naturally open to like getting help from people. It's just how I tackle things. But knowing that I have to like, confront something or deal with something, maybe it'll be different."* |
| | *"I think it's probably beneficial ... maybe... I don't really know because I don't have experience with it. But um, I can see it being like, helpful and keeping you grounded, I guess. Or like holding space, as they say."* |
| 1.2. Shifting attitudes towards touch following the psychedelic experience | *"I think the touch is a very, very important part of the experience. There were times there where I felt like, if I didn't have their hand to hold on to, that I would have been lost. Like I would have been gone. It's almost like a grounding connection"* |
| | *"I think everyone's so different. I'm not very comfortable with that kind of stuff, usually, but I still needed it. So probably most people will. I think just 'cause it's so like, intense an experience, maybe you do need that there. Just to let people know that there's someone there"* |
| | *"Yeah, it was it was comforting, but I wouldn't say that had it not happened it would have changed anything."* |
| | *"[The therapists] came over when I was … moving and looking kind of distressed … them just being right there, was, kind of, more helpful than necessarily the therapeutic touch."* |

*“No, I asked them not to [use therapeutic touch]... it's not like I'm one of those people that just doesn't like being touched. But it's more like I would have... been like conscious of say, my hand being clammy, because my hands are always clammy, or like, I just would've... I'm self-conscious”*

*“I didn't know if I'd need [touch]. But I did. [The dosing] turned out to be this super visceral experience … and I needed support at different points in time, and to have someone else support you, you know, the warmth of someone's hands, those types of things make a big difference. So it was really helpful. I think it's probably one of the more important parts of the process.”*

*“Yeah, it probably wasn't as effective during the placebo because I was basically just asleep the whole time ((laughs)) … Whereas, in the psilocybin, you're going through shifts - like you can see the person's going through an intense emotional experience. So a hand on the shoulder or holding your hand or something, you really feel that.”*

**Varied Experience and interpretation of touch**

| | |
|---|---|
| 2.1 The role of touch-facilitated connectedness for intense emotional experiences | *“I would have found that kind of sterile and like, standoffish if [the therapists] weren't allowed to [use touch]. If they were just like, sitting there observing, I'd feel a bit like a lab rat. And you know, that would just send your mind spinning when you're in the dosing session”*<br><br>*“At one point um, during that hard time when I was thinking about all my childhood stuff, I put out my hand and, and [Therapist One] took it and then [Therapist Two] took it at times as well. That was incredibly important to me because it made me feel connected. Like I wasn't alone.”*<br><br>*“At certain points [of the dosing] I felt really, really lonely. Um, and it wasn't like I felt physically isolated. It was just like ‘oh, I've been living in a way that means people don't know me’. Um, and then, there was a hand there. And it didn't really matter whose hand it was. It's just like, a sense of someone, whoever it is, here to reassure me that I'm not going to be by myself for this.”* |

| | |
|---|---|
| | *"My [dosing] experience was largely positive. I wasn't like struggling with stuff so I feel like [the therapists] didn't feel the need to come over and give me a hug or put a hand on a shoulder or anything like that. Maybe if I was going through something like that, I can see a situation where that would be really helpful. But yeah, that wasn't how it played out for me."* |
| 2.2 Bridging worlds: touch can affect the 'depth' of psychedelic experience | *"[The touch] was very therapeutic ((laughs)). Like ((deep inhale)) erm I don't know. I can't imagine having done it without that. They took turns holding my hand for like the whole time. Feeling that human contact, like connection, really helped keep me like, I don't know, stay grounded I guess, in that. Especially the first [dosing session]- it was just so, like, alien."*<br><br>*"In a way, you know, it's the way that they can show their support without necessarily interrupting the process. Because the only other alternative would be to be, you know, having a good old chat … and you don't want that … so somebody's just quietly holding your hand."*<br><br>*"Going through a psychedelic experience, you're putting yourself in a very vulnerable place where you don't have control, really. You can't really trust your sight anymore, you might just be vomiting and crying and you know, all those sorts of things. The world as you know it changes to a world where you don't really know and you don't have that control. And so because of that the touch is so important because it means that you feel safe. And feeling safe I think, is really important for creating the right 'set'"* |
| 2.3 Touch was considered therapeutic, independently of its support of psychedelic experience | *"It's just like, for me, [asking for support] is like a hard thing to do. So it was like, 'I don't want to do it' but then I was like, 'I probably should'. You know? Being part of this trial, you know, you want to change. So it's something that I find hard, but I need to be able to ask for help and stuff. So I think it was good to be able to do it"*<br><br>*"One of my intentions with the first [dosing session] that we'd spelled out was being um, open to, to help, and care. And so, I was really trying to practice that in the first one. And that involved asking for a hand, and that kind of thing."*<br><br>*"In the first dose ... I remember being quite disconnected - or not feeling able to really lean into that connection or ask for [touch]. I think it was a relatively internal experience. I think I'm quite used to doing things on my own and I kind of noticed that a bit. Even though I was feeling more comfortable being in the room with them than I'd imagined - like kind of facing them and showing emotion - I don't think I felt that connected to the touch even though I felt comfortable with it. But in the second session, I felt more comfortable to like ask for it, I think. After I'd just taken the medication I just let them know* |

| | |
|---|---|
| | *"I'm feeling quite anxious" and then they both like on either side, put a hand on my shoulder. And it felt like maybe I was able to connect with that a bit more.”*<br><br>*“I went to boarding school, right? For fucking six years, no one touched me. Like, do you know what I mean? Like, you know, you're in this environment with like- everything's really regimented and strict and you don't... like those loving touches, like your mom holding your hand, or hugging you, that stuff wasn't really there, right? So um, maybe that also [made touch] so helpful.”* |
| **Relational dynamics in delivery: trust and transparency** | |
| 3.1 The process of consent | *“There's some places I might not - like I don't like being touched on my feet or anything like that. Um, so it's good to go through that beforehand.”*<br><br>*“I was really worried about being touched, and the way that [Therapist One] described it was like "we're going to give you some headphones, and we're going to give you like eye shades, and then we'll check in on you by like giving you a gentle touch on the shoulder". And I was like "oh, my God, like this sounds like I'm going to have all my senses cut off and then just be laying there frozen, waiting for someone in the dark to like touch me suddenly" … And then we kind of had a chat about how I'm allowed to react, and saying the scary bits out loud is really helpful… I feel less anxious about it.”*<br><br>*“I think [what’s necessary for consent is] just like, what they did. Like go through it beforehand. What you're comfortable with, what you're not comfortable with … or, you know, what parts of your body and stuff.”*<br><br>*“They only touched me when I asked, so that helped a bunch.”* |
| 3.2 The role of trust and attunement in the experience of touch | *“I kind of said 'look, you know, you could touch my hand. Touch my shoulder. Whatever. But just read the room. Like, if I'm not feeling it, you should be able to pick it up, and if it's helping, keep doing it.’”*<br><br>*“I'm not a hand holder, and the thing is, when I went to dose one, I said to them, 'I'm completely fine, whatever you want to do, hold my hand, rub my forehead, whatever.' Um, but then dose one happened. And I literally did the turnover, ‘Don't come near me’.”* |

*“I kind of said, 'maybe I'm okay with like being stroked on the shoulder, and just by my female therapist', because erm yeah, when I have a doctor for instance, I always prefer a female … Erm in the first session, I was okay with, you know, being touched, it was all right. And so I said in the second dose that it can be either gender.”*

*“I can understand why therapists are so nervous, that's the wrong word, but so careful about it. … But for me [touch is] very, very important. And um I think one of the skills of the therapist is finding that exact place where correct boundary meets being completely open to being able to hold somebody. It's a very difficult call, I get it, but I think it's very important.”*
